# Gender-Specific Impact of Self-Monitoring and Social Norm Information on Walking Behavior Among Chinese College Students Assessed Using WeChat: Longitudinal Tracking Study

**DOI:** 10.2196/29167

**Published:** 2021-12-07

**Authors:** Yuepei Xu, Ling-Zi Yue, Wei Wang, Xiao-Ju Wu, Zhu-Yuan Liang

**Affiliations:** 1 CAS Key Laboratory of Behavioral Science Institute of Psychology Beijing China; 2 Department of Psychology University of Chinese Academy of Sciences Beijing China; 3 Brigham and Women’s Hospital Harvard Medical School Boston, MA United States

**Keywords:** self-monitoring, social norm, group identity, gender differences, mHealth, mobile health

## Abstract

**Background:**

Walking is a simple but beneficial form of physical activity (PA). Self-monitoring and providing information about social norms are the 2 most widely used “mobile health (mHealth)” strategies to promote walking behavior. However, previous studies have failed to discriminate the effect of self-monitoring from the combination of the 2 strategies, and provide practical evidence within Chinese culture. Some essential moderators, such as gender and group identity, were also overlooked.

**Objective:**

We aimed to investigate the effectiveness of social norm and self-monitoring interventions for walking behavior and assess the moderating effects of gender and group identity, which could guide optimal mHealth intervention projects in China.

**Methods:**

In 2 longitudinal tracking studies (study 1, 22 days; study 2, 31 days), Chinese college students wore trackers for at least 8 hours per day (MASAI 3D Pedometer and Xiaomi Wristband 2) to record their daily step counts in baseline, intervention, and follow-up stages. In each study, participants (study 1: n=117, 54% female, mean age 25.60 years; study 2: n=180, 51% female, mean age 22.60 years) were randomly allocated to 1 of the following 3 groups: a self-monitoring group and 2 social norm intervention groups. In the 2 intervention groups and during the intervention stage, participants received different social norm information regarding group member step rankings corresponding to their grouping type of social norm information. In study 1, participants were grouped by within-group member PA levels (PA consistent vs PA inconsistent), and in study 2, participants were grouped by their received gender-specific social norm information (gender consistent vs gender inconsistent). Piece-wise linear mixed models were used to compare the difference in walking steps between groups.

**Results:**

In study 1, for males in the self-monitoring group, walking steps significantly decreased from the baseline stage to the intervention stage (change in slope=−1422.16; *P*=.02). However, additional social norm information regardless of group consistency kept their walking unchanged. For females, social norm information did not provide any extra benefit beyond self-monitoring. Females exposed to PA-inconsistent social norm information even walked less (slope during the intervention=−122.18; *P*=.03). In study 2, for males, a similar pattern was observed, with a decrease in walking steps in the self-monitoring group (change in slope=−151.33; *P*=.08), but there was no decrease in the 2 social norm intervention groups. However, for females, gender-consistent social norm information decreased walking steps (slope during the intervention=−143.68; *P*=.03).

**Conclusions:**

Both gender and group identity moderated the effect of social norm information on walking. Among females, social norm information showed no benefit for walking behavior and may have exerted a backfire effect. Among males, while walking behavior decreased with self-monitoring only, the inclusion of social norm information held the level of walking behavior steady.

## Introduction

### Background

Walking is a simple but highly beneficial form of physical activity (PA) [1]. It can lower the risk of premature death [2] and prevent numerous chronic diseases such as obesity, type II diabetes, and cardiovascular disease [3]. In addition to providing reasonably accurate daily walking records [4], smartphones are also capable of additional functions such as tracking the number of steps walked. Such functionality has led to the use of mobile devices to promote a healthier lifestyle. Indeed, this concept of a mobile health (mHealth) strategy has become an increasingly popular public health intervention tool to promote walking behavior [5].

Providing social norm information and providing one's self-monitoring information (ie, a record of one’s behavior) are currently 2 of the most widely used strategies in mHealth. In self-monitoring interventions, pedometers or pedometer apps on smartphones (eg, Apple Health and Accupedo) provide users with daily step counts, which enable them to monitor and improve their own walking behavior [5-8]. Combined with this self-monitoring strategy, several leading online social media platforms (eg, Facebook and WeChat) have also implemented a social norm strategy to form an mHealth intervention for walking behavior [9,10]. The biggest difference between the 2 strategies is that the latter strategy allows users to view their own records and the walking records of their peers. By exposing users to a highly social norm sensitive context, users might be impacted by the perceived levels of others’ PA and might thereafter increase their own walking behavior [11-13]. Previous studies examining the effect of social norms have shown that there is still some debate regarding its veracity [14,15], and others have failed to consider the influence of some potential moderators [16]. Thus, it is still unclear as to whether a social norm strategy would be effective in promoting walking behavior.

In this study, we aimed to optimize mHealth walking interventions and provide more practical evidence about the effectiveness of interventions based on social norms. To address these aims, we implemented 2 longitudinal tracking studies in order to compare and isolate the mixed effects of social norm and self-monitoring interventions on promoting walking. In particular, we focused on 2 theoretically essential factors and ignored moderators regarding the influence of social norm interventions (group identity and gender).

### Self-Monitoring Intervention: Effect of One’s Own Walking Records

Self-monitoring refers to a systematic self-observation or recording of target behaviors (eg, walking and food intake) [17]. Based on the self-regulation theory, self-monitoring is an essential component of a successful self-regulation process, preceding self-evaluation and self-reinforcement [18]. Self-monitoring thus motivates individuals to make changes by stimulating them to focus, evaluate, and regulate the target behavior [17]. Several studies have shown that the strategy of self-monitoring plays a positive role in promoting a variety of healthy or healthier behaviors such as reduction in smoking [19] and improvements in diet [20].

Data from several studies suggest that a self-monitoring strategy, typically informing people how many steps they have walked, can be effective in boosting walking behavior [6-8]. For example, one study showed that by wearing a pedometer and setting a self-monitored goal of walking 10,000 steps each day, overweight women were able to increase their average walking steps by 85% (from 4972 to 9213 steps per day) [21]. A recent meta-analysis based on 6 interventions from 2005 to 2017 also showed that a self-monitoring strategy was associated with an increase in walking of up to 3090 steps per day in cardiovascular patients [22]. In a review of 88 intervention studies (N=18,804), Knittle et al concluded that self-monitoring is one of the most widely used strategies and represents an essential component for increasing both the intention of PA (*b*=0.30) and actual PA behavior (*b*=0.28) [23]. As a consequence, self-monitoring has generally been adopted as the centerpiece of most public health intervention programs [24,25].

However, there has been some controversy surrounding the effect of self-monitoring. For example, some researchers have argued that, despite its numerical effectiveness, the act of self-monitoring does not have a positive influence on walking behavior. Self-monitoring may also not guarantee high levels of exercise adherence, with rates of adherence shown to decline significantly over time [26].

More importantly, previous studies have paid little attention to exploring the mechanisms that underlie self-monitoring interventions in practice, thus limiting its application value and generalizability to other areas. For example, in order to optimize effectiveness, interventions have implemented a combination of strategies with self-monitoring included in a package of self-regulation processes [17], such as goal setting [21]. As a consequence, it is difficult to disambiguate the actual effect of self-monitoring from the mix of other potential effects. In addition, the majority of previous intervention studies have failed to consider the potential for certain demographic variables to exert a moderating effect on the relationship between self-monitoring and PA behavior [17]. Gender represents a key example whereby females may benefit more from a self-monitoring intervention and maintain an active lifestyle for longer than males [27].

### Social Norm Intervention: Effect of the Walking Information of Peers

Numerous theories of behavioral change, including the theory of planned behavior as an example, have listed social norms as an essential predictor of actual changes in behavior [28]. Previous studies have demonstrated that 2 types of social norms [29,30] are important for behavior change across areas ranging from reducing alcohol use [15,31] to reducing environmental damage [30]. These are descriptive norms, which refer to the perceived prevalence of target behaviors, and injunctive norms, which refer to perceived approval or disapproval of target behaviors by the society. Descriptive social norms may guide the behavior of individuals because of implied social proof that the behavior of the majority should be right, as the focus theory of normative conduct posits [30]. Injunctive norms may guide behavior because of a need by individuals to obtain social approval for meaningful relationships with other ingroup members [29,30].

In particular, the perception of social norms about PA was positively correlated with the actual PA level of individuals [13,32], and providing social norm information can even lead individuals to do more PA [12]. For example, compared with traditional online tracking or sending of promotional messages, interventions based on social norms, such as sharing the information of peers, led to participants spending more time on PA [10]. The typical use of descriptive norms, such as sending a message containing exercise information of colleagues, was more effective in increasing mild physical exercise than interventions that did not have any norm manipulation, such as sending general information about physical exercise and health [12]. McEachan et al analyzed 100 empirical studies from 1990 to 2010 (N=22,849) and found social norms to be a strong predictor for PA (mean correlation ρ=0.21, 95% CI 0.18-0.24) [33]. A series of meta-analyses [28,34] also revealed that changing one’s perception of social norms can produce a small to moderate effect (Cohen *d*=0.36) in increasing healthy behavior, including physical exercise.

However, the notion that social norm interventions are reliably effective has been complicated by some studies that have found its effect on behavioral change to be unstable or even negative [14,15]. For example, a meta-analytic review reported only a weak correlation (*r*=0.17, 90% CI −0.07 to 0.43) between social norms and PA [35]. The relationship between social norms and exercise intention may disappear after controlling for other relevant factors such as attitude to sports [36]. Indeed, a situation in which a desirable behavior is only a minority norm (eg, only a few people walk over 10,000 steps every day) or in which an undesirable behavior is the majority norm (eg, the majority only walk 2000 steps every day) may even lead to a backfire effect where an individual’s healthy behavior is reduced [37,38], suggesting that social norm interventions must be applied with caution. Even from a methodological level, since social norm interventions are generally combined with self-monitoring (ie, users may track their own walking and their peers’ walking at the same time [39]), it can become difficult to detect the pure effect of social norm interventions. Consequently, given that the implied factors that can lead to success or failure in these social norm intervention studies are unclear, researchers have argued that the likelihood of both type 1 and type 2 errors is high [38]. This has led some to suggest that social norm interventions should be dropped from further use [36]. Moreover, compared with correlational studies measuring subjective social norms, evidence-based interventions targeting social norms are still limited [36].

### Group Identity and Gender as Moderators of the Effects of Social Norm Interventions on PA

With the development of social norm theories, researchers have increasingly declared that the relationship between social norms and target behavior cannot be explained by a simple one-way causal model. Recent studies have attempted to explain the weak effect of social norm interventions on PA by investigating previously ignored moderators such as group identity [16]. However, most previous interventions simply provided social norm information by sending a message without investigating any other potential variables [10,12]. This may lead to a disparity between an up-to-date theory framework and previous practical interventions.

As one of the most widely used and effective theories in predicting and intervening in health behaviors, such as reducing alcohol intake [40] and promoting hand-washing [41], the theory of normative social behavior (TNSB) has proposed group identity as a factor moderating the effect of descriptive norms on behavior [42]. Group identity is defined as the degree to which an individual perceives similarity with the group and aspires to emulate group members [42]. The TNSB predicts that the effect of social norm interventions on behavioral change would be stronger when individuals perceive a higher similarity between themselves and the reference group [42], that is, social norms from a closer group work better at changing behaviors due to higher group identity, and individuals are more willing to perform the target behavior as an expression of group solidarity [42]. This moderating effect of group identity on the effect of social norm interventions can also be explained by the phenomenon of groupthink. Groupthink is more likely to take place in groups with higher identity and may put pressure on group members to conform to the social norms of the group [43]. Empirical studies support the prediction of the TNSB. For example, the closer individuals felt to a group, the closer their own behavior was to their perception of the social norms of that group [44]. Social norm interventions have been found to promote office colleagues with a high group identity to play more sports, while the effect was insignificant among college students who had a low group identity [12].

As an innate label, information regarding individuals with the same gender may feel more relevant and relatable. Therefore, according to the TNSB, because of a higher group identity, social norms within males or females (gender-consistent or gender-specific social norms) may have a greater impact on the behaviors of individuals [45]. For example, one study showed that perceived gender-consistent norms were stronger predictors of alcohol consumption than gender-inconsistent norms, and this was especially evident for females [46]. However, although previous studies have suggested that manipulating gender-consistent norms may be more beneficial in norm-based interventions [45,46], the availability of practical evidence is still limited and controversial. For instance, an intervention using gender-consistent norms did not show a better effect on reducing alcohol consumption than gender-inconsistent norms [47]. Gender may also interact with the effect of social norm interventions on target behaviors (different genders may have divergent reactions after receiving the same normative information). For instance, a social norm intervention regarding driving behavior may be effective for males but not females because of stronger normative pressure regarding this particular behavior in men compared to women [48]. On the other hand, females may be more sensitive to norms related to their body appearance than males due to their increased tendency to engage in appearance-related social comparison [49]. Even though these studies clearly suggest that the effectiveness of social norm interventions is strongly associated with gender, the importance of this factor has been largely overlooked in previous studies investigating mHealth interventions.

Moreover, the finding of systematic gender differences in the target behavior of this study (ie, PA) (see Pollard and Wagnild’s review [50]) calls for the need to consider gender as a moderating factor for the effect of social norm interventions on walking behavior. For example, walking has been observed to be a type of PA preferred by females [51], although consistent evidence has shown that males did more physical exercise than females [51-53]. According to evidence showing that self-regulation was the best predictor of PA in female college students but not males, females may be more sensitive to self-monitoring interventions [52]. Given that China currently has one of the widest gender gaps in the world [54], these gender differences within the Chinese cultural context may be more pronounced than those within other regions. In contrast to the commonly accepted gender-consistent attitude to sports, in China, males reported a more positive attitude to sports than females [55]. Compared with female college students, male students were also found to exercise more frequently, and they also showed both a higher participation rate and voluntary motivation to practice sports [56].

### Our Study

Since 2018, China has been the home of over 500 million mHealth users [57], making it an ideal region to evaluate the effects of different interventions. The use of smartphone pedometer apps has been found to be more favorable to traditional wearable pedometer devices in improving walking behavior [58]. Consequently, some social media platforms began to design their own pedometer plugins. WeChat, one of the most popular social media platforms with over 1 billion users in China [59], designed WeRun to provide daily step tracking and step ranking among the contacts of WeChat users. In addition to viewing rankings, users can give a thumbs-up or follow the step records of their contacts. With 15.3% of users taking advantage of its novel social functions, WeRun has become one of the most widely used features of WeChat since its launch in 2015. According to WeRun users, they use it to not only get data about their own exercise behavior, but also increase exercise frequency as well as interaction with friends [60]. As a consequence, WeChat represents a highly appropriate platform for investigating the effect of social norm interventions.

Although there are increasing numbers of apps, smart devices, and high-tech companies trying to promote PA among individuals through systems that utilize self-monitoring or social norms, little is known about the comparison of the effects of social norm interventions with self-monitoring interventions. The absence of substantial research investigating the underlying mechanisms of social norm interventions has obstructed the development of more personalized and optimized mHealth interventions. This disconnection between up-to-date social norm theories (eg, the TNSB) and antiquated intervention techniques may have weakened the potential for practical benefits from the existing evidence base.

Moreover, previous studies have suffered from some limitations, both methodologically and practically. For example, since most previous studies on social norms lacked a corresponding self-monitoring control group [39], the mixed-strategy approaches may have not only resulted in ambiguous comparisons of the effects of social norm and self-monitoring information on walking behavior, but also led to the collection of data characterized by low reliability and high error rates [38]. In addition, given that most mHealth interventions are specific to a certain app or device, the particular results of the corresponding interventions were device-specific and were thus not stable enough to be generalizable. In the context of mHealth, China is a rapidly developing country, but it is also suffering from a specific social issue in the shape of a wide gender gap. We have very limited understanding as to how gender and different mHealth interventions interact, and thus, there is no useful guidance as to how mHealth interventions in China can be optimized.

To address this, we aimed to answer the following 2 primary questions in this study: (1) In China, is an intervention based on social norms more effective than self-monitoring at promoting walking behavior? (2) Do gender and group identity moderate the effect of social norm interventions on walking? Specifically, we conducted 2 longitudinal tracking studies to compare the effects of self-monitoring and social norms on walking behavior. In study 1, we compared the effects of social norm and self-monitoring interventions on PA between genders by randomly assigning participants to a self-monitoring group, a PA-consistent intervention group (ie, similar levels of PA among group members), or a PA-inconsistent group (ie, varying levels of PA among group members). In study 2, after observing a gender-specific effect of social norm interventions on PA, we attempted to replicate the main finding of study 1 using a more precise measure of steps walked. We also assigned participants to a self-monitoring group, a gender-consistent intervention group (ie, providing social norm information that is gender-specific), or a gender-inconsistent intervention group (ie, providing social norm information that is not gender-specific). Our hypotheses were as follows: hypothesis 1 (H1), in China, providing social norm information, in the form of step ranking, can promote walking more effectively compared with self-monitoring; hypothesis 2 (H2), gender will moderate the effect of social norm interventions on the promotion of walking, and the walking behavior of males will be more impacted by social norms; and hypothesis 3 (H3), group identity will moderate the effect of social norm interventions on the promotion of walking, and a higher sense of group identity will strengthen the effect of social norm interventions. Furthermore, H3 had the following 2 parts: H3a, an intervention using PA-consistent norms will have a larger effect on walking behavior than that using PA-inconsistent norms; H3b, an intervention using gender-consistent norms will have a larger effect on walking behavior than that using gender-inconsistent norms.

## Methods

### Participants

In both studies, we recruited graduate students from the University of Chinese Academy of Sciences, Beijing, China as our participants by sending advertisements during university courses (744 students in study 1, and 986 students in study 2). To avoid the possible confounding effect of walking habits or previous mHealth app experience, all recruited students completed a 5-minute online screening and customized sports habit questionnaire. The inclusion criteria were as follows: (1) used WeRun or other walk-related mHealth apps less than twice a week; (2) not a member of any WeChat sports group; (3) self-reported being mentally and physically healthy; (4) consented to participate in the entirety of the experiment. Next, according to their self-reported sports habits, participants were labeled as either low PA (sports fewer than twice a week and run a total distance of less than 5 km per week) or high PA (sports more than three times per week and run a total distance of more than 10 km per week).

Given that the motivation of this study was to promote walking behavior among the low-PA sample, we only selected low-PA students as our formal participants (high-PA students were only recruited in study 1 to set up the PA-inconsistent group, but were not included for further analysis). We preset our sample size to at least 40 participants in each group. Thus, we included a total of 127 low-PA participants in study 1 (with another 42 high-PA students), while 182 low-PA participants were recruited for study 2.

This study was approved by the Institutional Review Board of the Institution of Psychology, Chinese Academy of Sciences, and all participants took part in the study voluntarily and completed written informed consent. To record the number of steps walked, all participants were asked to wear step trackers (MASAI 3D Pedometer, study 1) or smart bands (Xiaomi Wristband 2, study 2) during the entire experimental period. After the study, participants were allowed to keep their step trackers (and approximately US $18 in study 1) or smart bands as payments for participation.

### Design and Procedures

#### Study Design

**Figure 1:**
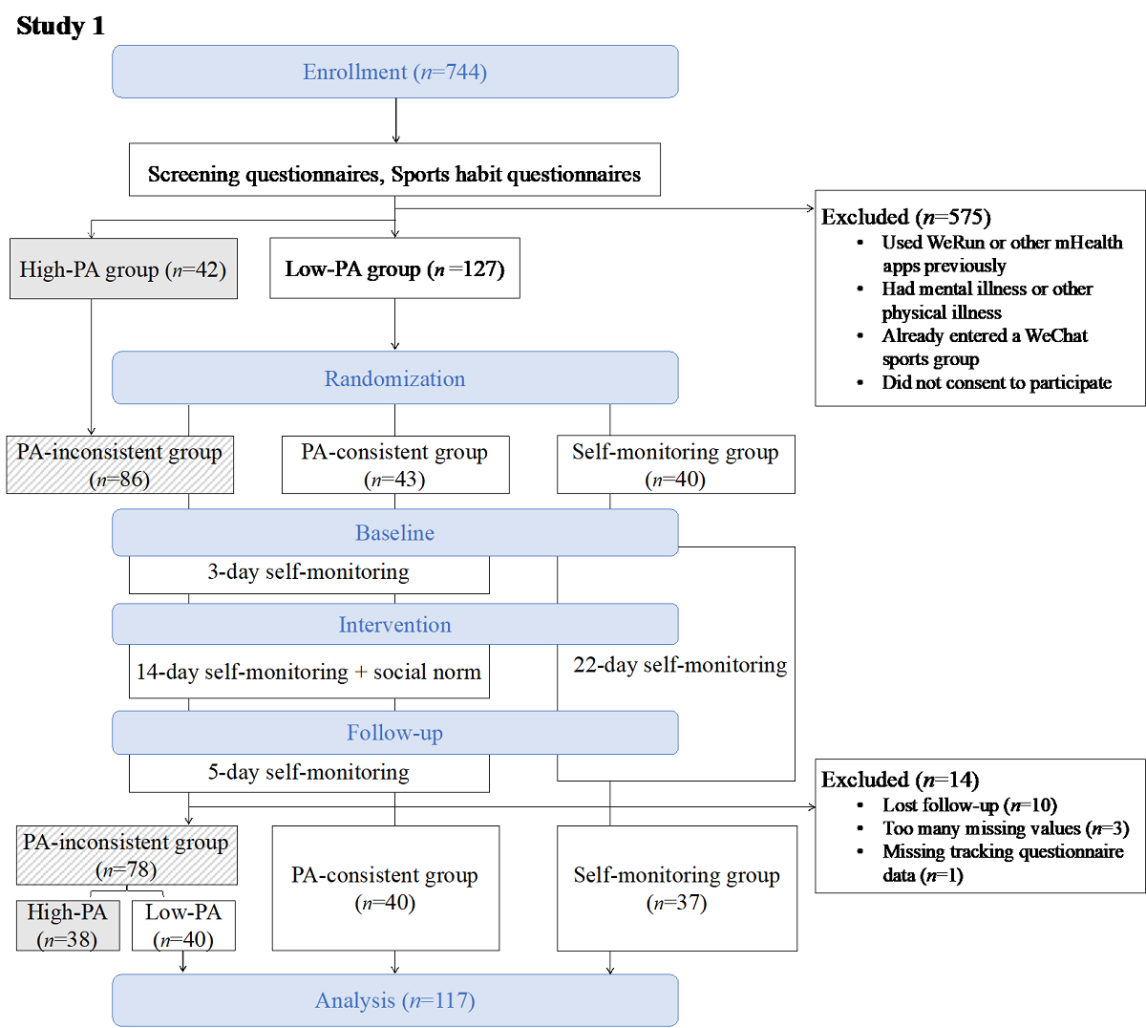
Flow diagram for study 1. The diagram shows the complete experimental procedure including enrollment, randomization, and intervention. All participants (valid n=117) in 3 groups were tracked for 22 days. PA: physical activity.

**Figure 2:**
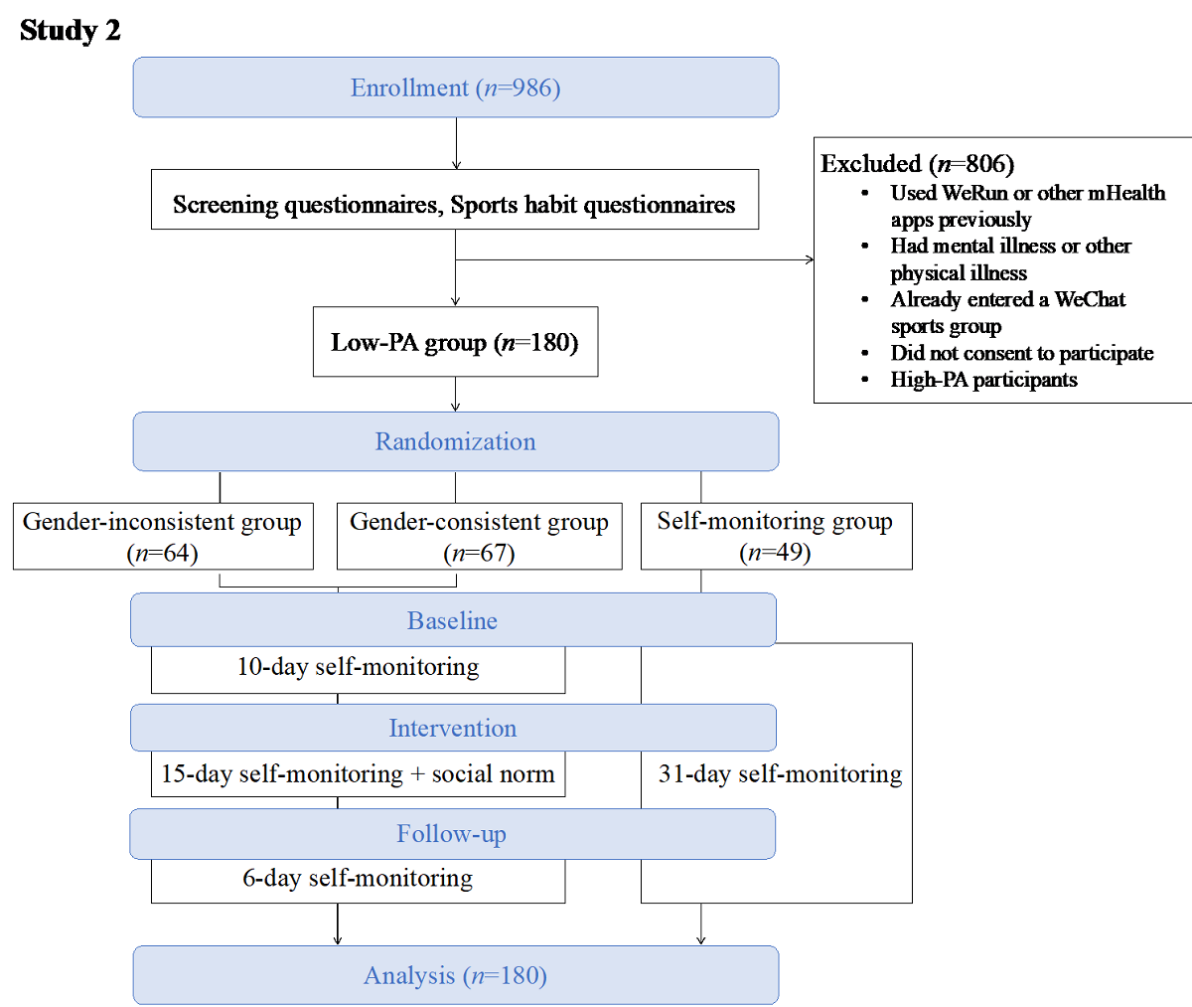
Flow diagram for study 2. The diagram shows the complete experimental procedure including enrollment, randomization, and intervention. All participants (valid n=180) in 3 groups were tracked for 31 days. PA: physical activity.

To compare the effects of self-monitoring and social norms, both studies consisted of 3 stages (baseline, intervention, and follow-up; Figure 1 and Figure 2), with slight variations in the length of each stage. Study 1 was conducted from April to May 2017 and consisted of 22 days, with 3, 14, and 5 days in each stage, respectively. Study 2 was conducted from October to November 2017 and consisted of 31 days, with 10, 15, and 6 days in each stage, respectively. All participants were required to wear the trackers during the entire experiment and were able to check their step counts at any time in order to monitor their own walking behavior.

To compare the effects of self-monitoring and social norms, we randomized all participants in both studies into 3 groups according to the interventions they received, namely, a self-monitoring intervention group and 2 social norm intervention groups. Participants in the 2 social norm intervention groups received social norm information from experimenters during the intervention stage, whereas participants in the self-monitoring intervention group did not receive any extra message during the experimental stages.

To test the moderating effect of group identity on the effect of social norm interventions, we aimed to compare the effects of providing social norm information among groups that should theoretically have a stronger sense of group identity (ie, PA-consistent and gender-consistent groups) against those that should theoretically have a weaker sense of group identity (ie, PA-inconsistent and gender-inconsistent groups). In particular, in study 1, we randomly assigned participants to either the self-monitoring group or 2 social norm intervention groups (the PA-consistent intervention group and PA-inconsistent intervention group). In the PA-consistent intervention group, all participants had a low PA level, while in the PA-inconsistent intervention group, half of the participants had a low PA level and half had a high PA level. In study 2, we randomly assigned participants to either a self-monitoring group or 2 social norm intervention groups (the gender-consistent intervention group and gender-inconsistent intervention group). In the gender-inconsistent (but PA-consistent) intervention group, participants received general gender-mixed social norm information as in study 1 (ie, gender-mixed information simply showing the rank of each member in the WeChat group), while in the gender-consistent (and PA-consistent) intervention group, participants received 2 gender-separate social norm information sets (ie, 2 gender-specific information sets showing the rank of each female student among all the female members and each male student among all the male members). Through this design, participants in the PA-consistent group in study 1 and gender-consistent group in study 2 will theoretically have a stronger sense of group identity than participants in the other groups.

Except for high-PA participants in study 1, all low-PA participants in both studies were randomly allocated to 1 of the 3 groups according to randomly generated numbers. All participants were single-blind to the nature of our experimental design during the entire experimental period.

#### Intervention Procedures

During the whole experimental period (including baseline, intervention, and follow-up), each participant was able to monitor his/her own steps by checking the trackers at any time. In addition, in order to send social norm information within the 2 social norm intervention groups of both studies, during the intervention stage, we set up separate WeChat groups for participants within each intervention group. Participants in the WeChat groups were asked to send a picture of their step trackers (study 1) or smart bands (study 2) before sleep every day. The next day at 10 AM, experimenters sent a personalized step ranking to each WeChat group member regarding their performance from the previous day.

To ensure that participants received the daily step ranking and paid sufficient attention to it, they were asked to finish an online daily tracking questionnaire containing 3 questions about step rankings (eg, How many steps did the person rank first in the group walk yesterday?) and their step tracker wearing habits (eg, When did you start to wear the tracker today?).

#### Social Norms and Other Measurements

To measure social norms as our secondary outcomes, participants also completed a social norm questionnaire on the last day of each stage (total of 3 times) that was adapted from a previous study [61]. The injunctive social norm questionnaire (6 items) asked participants to judge on a 7-point Likert scale (1, not agree at all to 7, totally agree) whether they agreed on some positive statements about playing sports (eg, most successful people have habits associated with playing sports). The descriptive gender norm questionnaire (2 items) measured participants’ descriptive male gender norms on walking (estimation of the percentage of male students in the same university walking over 6000 steps) and descriptive female gender norms on walking (estimation of the percentage of female students in the same university walking over 6000 steps). Moreover, participants were asked to self-report some demographic information, including their age, subjective health (on a 5-point Likert scale; 1, very unhealthy to 5, very healthy), height (m), and weight (kg), and BMI was calculated (weight/height^2^).

### Data Cleaning

Consistent with previous studies [39], we deleted data points for the following reasons: participants failed to answer all the daily tracking questions, device wear time was shorter than 8 hours (in study 1), or step count was less than 1000. In study 1, 4.72% of data points of step counts were missing, and in study 2, 0.79% of data were missing. Days with nonmissing data from the same participant were still included in the final analyses.

In study 1, we excluded a total of 14 participants from all analyses for the following reasons: 10 were lost in the follow-up stage, 1 did not answer the tracking questionnaire during the intervention stage, 1 had no valid baseline step data, and 2 lacked sufficient intervention step data (more than five missing values). The final valid sample included 155 participants (75 males and 80 females; mean age 25.75 years, SD 1.27 years; age range 23-33 years), of which 117 participants were from low-PA groups. In study 2, 2 participants quit the experiment halfway through and were thus excluded from all analyses. This left a valid sample of 180 participants (92 males and 88 females; mean age 22.60 years, SD 1.16 years; age range 20-30 years).

### Statistical Methods and Data Analysis

#### Baseline Comparison

In order to ensure the homogeneity of all the experimental groups, we conducted analysis of variance (ANOVA) to compare participant characteristics (age, subjective health level, and BMI) and baseline average step counts between the 3 groups. The homogeneity of variance was tested and satisfied. The significant factor (BMI in study 1) was included as a covariate in the analyses for the main outcomes.

#### Analysis for Social Norms

In order to compare changes in social norms (injunctive norms, male descriptive norms, and female descriptive norms) among groups across different experiment stages, we used a linear mixed-effects (LM) model with the stage (baseline, intervention, and follow-up) as a within-group factor, group and gender (male and female) as between-group factors, BMI in study 1 as a covariate, and the participant as a random intercept term in the model.

#### Analysis of Walking Step Data

Walking step data in our studies had 2 key features. First, there were 3 different stages (baseline, intervention, and follow-up) in the entire experiment. Second, each stage consisted of a segment of repeatedly measured daily step counts from each participant (in study 1, a total of 22 daily step counts, while in study 2, a total of 31 daily step counts). Given these features, to better capture the variability of participants’ daily step counts, both at the individual level and the trend during each stage, we applied a piece-wise linear mixed-effects (PLM) model. Compared with a traditional simple linear model with a constant slope, a piece-wise linear model implements several segmented regression lines with different slopes, and thus is suitable to perform staged and especially time-segmented prediction [62]. Meanwhile, the mixed effects of the PLM take into account the individual differences during repeated measurements in each experiment stage [63].

The PLM model for walking step data was conducted for each gender separately. The predictors of the model were group, time, and their interactions, with BMI as a covariate in study 1 and the participant as a random intercept term in the model. The predictor “time” was defined by combining the continuous variable “day” during the entire observation period (22 days for study 1; 31 days for study 2) and the dummy categorical variables to reflect “stage” (defined as baseline days 1-3, intervention days 4-17, and follow-up days 18-22 for study 1; and baseline days 1-10, intervention days 11-25, and follow-up days 26-31 for study 2) to allow different linear trends over different stages. The resulting 3 independent slopes represented the different changes of each group from baseline to the intervention stage, as well as from the intervention stage to the follow-up stage. The model also allowed different intercepts for each participant to control for possible variability in individuals’ overall levels of steps. In this study, as participants in all 3 groups could self-monitor their steps during the entire experimental period (baseline, intervention, and follow-up), the changes in the slopes from baseline to the intervention stage in the 2 social norm intervention groups will represent the extra effects of social norm information on walking (isolated from the effect of self-monitoring information in the entire experimental period).

Next, to more closely examine the intervention effect, we also created an LM model by only including the data from the intervention stage (14 days in study 1 and 15 days in study 2). The predictors of the model were group, day, and their interactions, with BMI as a covariate in study 1 and the participant as a random intercept term in the model. Then, by comparing the slopes of “day,” we were able to compare the effects on walking performance between providing self-monitoring alone and providing social norm information and self-monitoring.

Considering that the ranking position may impact the effect of social norms, we also added ranking position as a new covariate in the PLM. The results remained the same with the ranking position as a covariate (see detailed information in Table S1 in Multimedia Appendix 1 and Table S1 in Multimedia Appendix 2).

We performed all data analyses using R 4.0.3 (R Project for Statistical Computing). We set the level of significance for all analyses to .05. A *P* value between .05 and .10 was considered marginally significant.

## Results

### Study 1

#### Participant Characteristics

Detailed information about participant characteristics is provided in Table 1. ANOVA showed that, for both males and females, control variables were not significantly different among groups, except for BMI (*P*=.03 and .03 for males and females, respectively). Consistent with our grouping criterion that the high-PA group would walk more, we observed a significantly higher baseline step count (male high-PA group: mean 11,775, SD 3970; female high-PA group: mean 10,860, SD 3879; *P*<.001), compared with the mean of the 3 low-PA groups. These results indicate that, except for BMI, the 3 low-PA groups were basically homogenous for both males and females. Thus, we added BMI as a covariate in all subsequent analyses for study 1.

**Table 1:** Participant demographic characteristics and baseline steps in study 1.

Variable	Male (N=54)				Female (N=63)			
	Self-monitoring (n=15)	PA^a^-consistent intervention (n=21)	PA-inconsistent intervention (n=18)	*P* value	Self-monitoring (n=22)	PA-consistent intervention (n=19)	PA-inconsistent intervention (n=22)	*P* value
Age (years), mean (SD)	25.87 (1.25)	25.57 (1.03)	25.72 (1.02)	.72	25.32 (0.89)	25.42 (0.96)	25.77 (1.02)	.27
Subjective health, mean (SD)	3.47 (0.92)	3.38 (0.86)	3.67 (0.69)	.55	3.32 (0.72)	3.32 (0.58)	3.05 (0.72)	.33
BMI, mean (SD)	22.54 (2.13)	22.86 (3.58)	20.51 (1.95)	.03	20.23 (2.29)	20.76 (1.23)	19.18 (1.83)	.03
Baseline steps, mean (SD)	8687 (3759)	7670 (3130)	8634 (3105)	.57	8356 (3717)	7527 (3407)	7623 (2376)	.66

^a^PA: physical activity.

#### Changes in Social Norms

The LM model results (Table S1 in Multimedia Appendix 3) showed a significant main effect of stage on descriptive gender norms (male gender norm: *F*_2,222_=28.70; *P*<.001; female gender norm: *F*_2,222_=29.16; *P*<.001) and injunctive social norms (*F*_2,222_=5.23; *P*=.01). In particular, during the experimental stages, the descriptive gender norm perceptions among all 3 groups increased progressively (*P*<.001). Participants’ estimations about the percentage of male students walking over 6000 steps increased from a mean of 40.84% (SD 20.69%) to 49.80% (SD 18.62%) and then to 55.97% (SD 17.56%). Moreover, participants’ estimations about the percentage of female students walking over 6000 steps increased from a mean of 31.83% (SD 19.32%) to 41.31% (SD 18.56%) and then to 47.21% (SD 17.85%). Among female students, injunctive social norm perceptions also increased significantly (*P*=.004) from baseline (mean 5.35, SD 0.92) to the follow-up stage (mean 5.66, SD 0.74). Surprisingly, however, we failed to observe a significant group difference in changes in social norms. This suggests that the self-monitoring intervention alone may be capable of leading people to perceive a higher level of social norms about walking.

#### Effect of the Social Norm Intervention on Step Count

**Figure 3:**
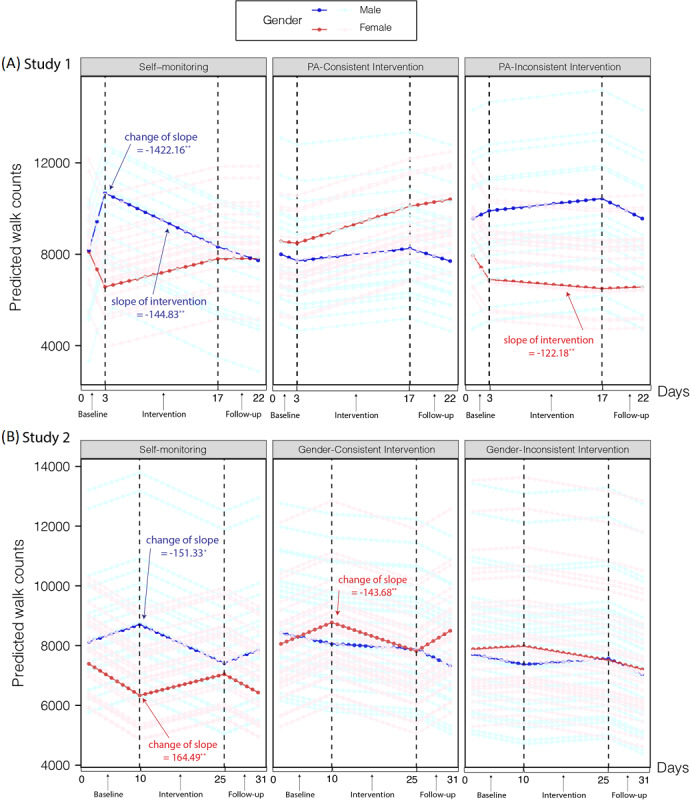
Step counts (piece-wise linear mixed-effects model predicted) of the self-monitoring and 2 social norm intervention groups in study 1 (A) and study 2 (B). Each polyline represents a participant. One highlighted polyline is used for each group and gender (male, blue line; female, red line). All (marginally) significant changes of slope and slope of the intervention are labeled. *P<.10 but >.05, **P<.05.

As shown in Table 2 and Figure 3A, the PLM model showed separated patterns for genders with regard to step count. For males in the self-monitoring group, we detected a significantly negative change in slope over time from baseline to the intervention stage (*P*=.02), suggesting that they walked less after the 3-day baseline. For males within the 2 social norm intervention groups, walking did not change during the whole experiment. For females, however, no significant slopes or changes in slopes over time were found, suggesting that the walking behavior of females did not change in any of the 3 groups during the whole experiment. BMI was also a significant predictor in females but not males. Specifically, a higher BMI predicted more steps taken by females (slope=328.13; *F*_1,59.23_=6.21; *P*=.02), a pattern which was not repeated by males (slope=101.91; *F*_1,49.92_=0.57; *P*=.45). These results suggest that higher BMI motivated females, but not males, to walk more steps.

These results partially supported H1 and H2, suggesting that the effect of social norm interventions is gender-specific. For males in the self-monitoring group, the promoting effect of self-monitoring on walking was short-lived. Although the social norm intervention did not robustly increase walking in males, the extra social norm information, regardless of its PA consistency, could at least prevent their walking steps from decreasing. For females, self-monitoring showed a stable but nonsignificant effect on walking behavior, and we found no evidence that social norms worked better than self-monitoring.

**Table 2:** Results of the piece-wise linear mixed-effects model in study 1 across all experimental stages.

Gender and group		Intercept	Baseline		Intervention		Follow-up		Conditional *R*^2^
			Slope	*P* value	Change in slope	*P* value	Change in slope	*P* value	
**Male (N=54)**									0.27
	Self-monitoring (n=15)	8169.98	1254.20	.03	−1422.16	.02	48.66	.84	
	PA^a^-consistent intervention (n=21)	7930.21	−142.53	.77	181.10	.71	−148.91	.46	
	PA-inconsistent intervention (n=18)	9267.26	166.57	.75	−128.39	.82	−213.44	.32	
**Female (N=63)**									0.15
	Self-monitoring (n=22)	8082.79	−766.83	.14	854.63	.12	−84.81	.68	
	PA-consistent intervention (n=19)	8181.32	−37.13	.95	152.45	.79	−54.04	.81	
	PA-inconsistent intervention (n=22)	8118.16	−525.53	.30	496.86	.35	42.54	.84	

^a^PA: physical activity.

#### Comparing the Effects of Self-Monitoring and Social Norms on Step Count

Results from the LM model at the intervention stage indicated (details in Table 3) a clear distinction between genders in terms of sensitivity to self-monitoring and social norm interventions. For males, the interaction between time and group was marginally significant (*F*_2,682.11_=2.48; *P*=.09). In the self-monitoring group, we observed a significantly negative slope (*P*=.04), suggesting that steps taken by males in this group decreased during the intervention stage, while both the PA-consistent and PA-inconsistent interventions arrested this decreasing trend. For females, however, the interaction between time and group was significant (*F*_2,793.57_=3.39; *P*=.03). A significantly negative slope (*P*=.03) for the PA-inconsistent intervention group also suggested that the PA-inconsistent intervention led to a decrease in steps walked during the intervention stage. No such effect for females in either the self-monitoring or PA-consistent intervention group was found. Consistent with findings from the PLM model, a higher BMI in females was a significant predictor of more steps walked (*F*_1,59.35_=4.22; *P*=.04). Again, this pattern was not found in males (*F*_1,49.91_=0.75; *P*=.39).

**Table 3:** Results of the intervention-focused linear mixed-effects model in study 1.

Gender and group		Intercept		Slope	SE	*P* value	Conditional *R*^2^
**Male (N=54)**							0.31
	Self-monitoring (n=15)	8448.02		−144.83	71.43	.04	
	PA^a^-consistent intervention (n=21)	8068.35		37.04	60.92	.54	
	PA-inconsistent intervention (n=18)	9520.74		48.70	65.76	.46	
**Female (N=63)**							0.21
	Self-monitoring (n=22)	7800.66		81.14	57.49	.16	
	PA-consistent intervention (n=19)	7973.10		32.73	61.70	.60	
	PA-inconsistent intervention (n=22)	8141.32		−122.18	57.44	.03	

^a^PA: physical activity.

Taken together, these results partially supported H1 and H2, with several restrictions. For males in the self-monitoring group, self-monitoring only was not able to exert any long-term effect since steps decreased significantly, while for males in the 2 social norm intervention groups, steps were kept unchanged by the provision of social norm information. In contrast, for females, social norms did not work more effectively than self-monitoring. The hypothesized moderating effect of group identity (H3a) was supported, but it also interacted with gender. In particular, for females, PA-inconsistent social norms had a negative effect on walking behavior.

In summary, these results preliminarily confirmed H2 and H3, revealing a gender-specific moderating effect of group identity on social norms. Given the finding that gender did play an essential role in moderating the effect of the social norm intervention and considering the reliability of our results, we conducted study 2 to replicate this study. Moreover, we modulated gender consistency to further examine the effect of group identity.

### Study 2

#### Participant Characteristics

Detailed information of participant characteristics can be found in Table 4. ANOVA showed that, for both males and females, these baseline characteristics were not significantly different among groups (Table 4), indicating a fair homogeneity for the 3 experimental groups among both males and females.

**Table 4:** Participant demographic characteristics and baseline steps in study 2.

Variable	Male (N=88)					Female (N=92)			
	Self-monitoring (n=23)	Gender-consistent intervention (n=33)	Gender-inconsistent intervention (n=32)	*P* value	Self-monitoring (n=26)		Gender-consistent intervention (n=34)	Gender-inconsistent intervention (n=32)	*P* value
Age (years), mean (SD)	22.57 (1.20)	23.06 (1.50)	22.88 (1.29)	.41	22.58 (0.95)		22.24 (1.02)	22.28 (0.63)	.29
Subjective health, mean (SD)	3.30 (0.82)	3.42 (0.61)	3.41 (0.50)	.77	3.35 (0.56)		3.29 (0.72)	3.44 (0.56)	.65
BMI, mean (SD)	22.40 (3.56)	21.71 (2.52)	21.41 (3.72)	.54	19.89 (2.59)		19.42 (1.16)	20.54 (2.52)	.14
Baseline steps, mean (SD)	8737 (2279)	8750 (2211)	8012 (2647)	.39	7358 (1518)		8099 (1732)	8307 (2788)	.22

#### Changes in Social Norms

Similar to study 1 (Table S1 in Multimedia Appendix 4), for the 2 types of descriptive gender norms, LM model results showed a significant main effect of the stage (male gender norm: *F*_2,348_=13.80; *P*<.001; female gender norm: *F*_2,348_=22.39; *P*<.001). Post-hoc analysis showed that both descriptive male and female norms increased (*P*<.001) as the experiment progressed (from baseline to intervention to follow-up). However, in terms of injunctive social norms, LM model results showed no significant main or interaction effect. These findings indicate that injunctive norms in participants across all 3 groups did not change significantly with the experiment process.

In contrast to study 1, we found that females perceived higher descriptive gender norms than males. When asked “What is your estimation of the percentage of male/female students in the same university walking over 6000 steps?” (descriptive male/female gender norms), female participants estimated that 52.53% (SD 20.68%) of male students and 40.13% (SD 19.76%) of female students would walk over 6000 steps, while male participants estimated that 42.00% (SD 19.98%) of male students and 33.99% (SD 19.06%) of female students would walk over 6000 steps. In addition, there was a significant interaction between group and gender (*P*=.01) for the descriptive male norm. Post-hoc analysis showed that there was no significant group difference for female participants (adjusted *P*>.10). However, for male participants, the gender-consistent group had significantly higher norms than both the self-monitoring (adjusted *P*=.01) and PA-consistent groups (adjusted *P*=.09). In detail, males in the gender-consistent group estimated that 48.29% (SD 19.91%) of male students walked over 6000 steps, while male participants in the self-monitoring and gender-inconsistent groups estimated that 35.54% (SD 19.51%) and 40.16% (SD 18.67%) of male students walked over 6000 steps, respectively. In contrast, their estimations for female students were not significantly different.

Again, these results indicated that descriptive gender norms kept increasing for both genders as the experiment progressed from baseline to intervention to follow-up. We found that the gender-consistent manipulation was effective in influencing the gender norm perceptions of males but not females.

#### Effect of the Social Norm Intervention on Walking Behavior

Results of the PLM model in Table 5 and Figure 3B replicated the finding in study 1 of a different effect of self-monitoring and social norms for each gender. For males, a self-monitoring–only intervention led them to walk less after baseline, while additional social norm information may help keep their walk steps unchanged. Only males in the self-monitoring group walked less after baseline as indicated by a marginally significant negative change of slope over time from baseline to the intervention stage (*P*=.08). On the other hand, males in the 2 social norm intervention groups did not show any change in walking behavior during the entire experiment.

However, in contrast to study 1, we found a backfire effect of gender-consistent social norms among females. Females in the self-monitoring group walked more after baseline, and the slope changed in a positive direction over time from baseline to the intervention stage (*P*=.03). However, females in the gender-consistent group walked less compared to baseline (ie, the backfire effect), and the slope changed in a negative direction over time from baseline to the intervention stage (*P*=.03). These results again suggested that social norms might help males to maintain long-term walking behaviors, while they do not provide the same benefit and could even exert a negative effect (gender-consistent social norms) on the walking behavior of females.

**Table 5:** Results of the piece-wise linear mixed-effects model in study 2 across all experimental stages.

Gender and group		Intercept	Baseline		Intervention		Follow-up		Conditional *R*^2^
			Slope	*P* value	Change in slope	*P* value	Change in slope	*P* value	
**Male (N=88)**									0.25
	Self-monitoring (n=23)	8159.99	64.89	.30	−151.33	.08	160.51	.20	
	Gender-consistent intervention (n=33)	8518.26	−39.97	.45	28.29	.69	−81.14	.44	
	Gender-inconsistent intervention (n=32)	7839.48	−37.16	.49	50.25	.49	−100.37	.35	
**Female (N=92)**									0.22
	Self-monitoring (n=26)	7303.92	−117.33	.04	164.49	.03	−148.91	.19	
	Gender-consistent intervention (n=34)	8014.41	79.70	.11	−143.68	.03	177.38	.07	
	Gender-inconsistent intervention (n=32)	7970.47	10.94	.83	−42.36	.54	−19.19	.85	

#### Comparing the Effects of Self-Monitoring and Social Norms on Walking Behavior

We did not find any significant slopes in the intervention-focused model. Detailed results from the LM model over the intervention period are shown in Table S1 and S2 in Multimedia Appendix 2. These results partially supported H1 and H2, and essentially replicated results from study 1, suggesting that the effect of social norm interventions on walking is only weak among males but not females. For males, walking behaviors that were driven by self-monitoring only began to decrease slightly after 10 days, while for those receiving social norm information, regardless of whether this information was gender-specific (ie, in the gender-consistent group) or not (ie, in the gender-inconsistent group), the walking behaviors remained unchanged. However, contradicting H3b, for females, gender-consistent social norms produced a backfire effect on the promotion of walking, suggesting that without these social norms, self-monitoring only may have led to females walking more.

## Discussion

### Principal Findings

Through 2 longitudinal tracking studies in a sample of Chinese college students, we made several findings. First, gender moderated the effect of the social norm intervention on walking. For males, the effect of the self-monitoring–only intervention was short-lived, while the addition of social norm information, regardless of its PA consistency or gender consistency, was able to keep the walking behavior unchanged. For females, we found no evidence to show that social norms performed better than self-monitoring. Second, an additional higher level of group identity in females did not consistently guarantee an improved effect of the social norm intervention on PA. With PA-inconsistent social norms (study 1) and gender-consistent social norms (study 2), the results suggested even a backfire effect on walking steps among females.

### Gender-Specific Effectiveness of Social Norm Interventions for Walking

Most previous intervention studies considered self-monitoring and social norms as 2 independent and effective strategies for promoting PA [7,8,12]. Little attention was paid to how these 2 strategies might compare in terms of effectiveness. In our studies, we combined these 2 strategies (self-monitoring–only intervention vs social norms and self-monitoring intervention) in order to directly compare their effects. Consistent with previous work [10,12,26], both studies consistently revealed that the effects of self-monitoring on PA (especially among males) were short-term (at least no longer than 3 or 10 days). We found that, among males, the addition of sharing social norms could outperform the effects of self-monitoring only. These results were partially consistent with those of Rote et al [9]. They reported that young women who shared PA information with Facebook group members were able to increase their PA more than those who only used a self-monitoring strategy. These results differed from our results in terms of gender.

As a meta-analysis [23] has previously suggested, our results revealed a notable separation in the patterns of PA outcomes according to gender. Specifically, for males, with self-monitoring as a baseline condition, we found that the effect of the intervention with social norm information can keep walking unchanged among males. However, for females, we found no evidence that social norms performed better than self-monitoring. A reasonable explanation for gender differences in the effect of a social norm intervention is that a relatively weak self-monitoring effect among males may leave more potential for a social norm intervention to produce a protective effect. Previous studies suggested that male students may be less self-disciplined [64] and less likely to use self-regulation strategies [65] than females. As a result, both of our studies showed a marginally significant decrease in walking in males after the baseline stage (in study 1, the change of slope over time was −1422.16; *P*=.02; in study 2, the change of slope over time was −151.33; *P*=.08), revealing a short-lived effect of self-monitoring among males when no social norm information was provided. Thus, as the effect of self-monitoring began to fade over time [66], additional social norm information was able to encourage males to perform social comparisons between themselves and their peers, thus motivating them to pursue a better rank position by walking more. For females, however, the self-monitoring intervention tended to benefit them more significantly, as they represent a more self-disciplined group [64]. A previous meta-analysis also concluded that the effect size of wearing a pedometer is much greater for females (95% CI 0.64-0.97) than males (95% CI −0.18 to 0.79) [66]. Therefore, a simple wearable pedometer may already lead highly motivated females to walk more, thus making it more difficult for other interventions to produce an additional enhancement or further increase the PA of females. This may be the reason why social norm information or supplemental PA intervention strategies [67], other than self-monitoring, work better for males than females.

An alternative explanation may be related to a counterproductive cultural stereotype, prevalent in Chinese culture, that females should be less active and petite than males [68]. From this perspective, constant PA may be deemed as an obstacle to adhering to such a stereotype regarding femininity, which may consequently discourage some female college students from engaging in PA. This explanation is supported by our results that the descriptive female gender norms were consistently lower than the male gender norms in both studies. Taking study 2 as an example, participants estimated that only 37.13% (SD 19.65%) of female students would walk over 6000 steps; however, for male students, the estimation was 47.38% (SD 20.99%). Still, these stereotypes of walking gender difference may only be effective as a self-serving bias for males. In study 2, the actual within-group rankings of females were even better than those of males; 53% of the highest quarter of the step ranking involved females in the gender-inconsistent intervention group. As a result, a relatively high level of individual PA may be perceived as a quality outside the “normal” confines of femininity, thus deviating from the traditional female stereotype, or, even worse, it could be considered as having an atypical female behavior. Previous studies have shown that a high level of PA may expose females to feelings of social pressure [69] and being stigmatized [70]. Thus, even if a female could have a similar step ranking to a male, the psychological meaning of ranking (the social norm information) might be different because, among females, having a high PA level might be perceived as an outside-gender group behavior rather than a within-gender group social norm. However, males may be motivated purely by the desire to pursue a better rank position, since walking more or having high PA is consistent with their own gender stereotype and set of social expectations. Thus, ranking as a motivational device was only effective for males, and in light of cultural expectations regarding femininity, no positive effect of social norm information was found among females. Future studies may benefit from comparing the effects of self-monitoring and social norm interventions on PA within different cultural backgrounds, or perhaps examining gender stereotypes unique to the cultures of participants’ countries.

It should be pointed out that, based on the current evidence, providing social norm information might have no more than a protective effect. Since neither study revealed a significantly positive change in slope in any social norm intervention group during the intervention stage, the provision of social norm information did not produce any additional enhancement in PA. Among males, however, both studies suggested that self-monitoring on its own may lead to a reduction in steps walked following the baseline stage. The addition of social norm information was, at least, shown to hold the level of PA steady in males, which can be considered as a protective effect.

### A Higher Group Identity Does Not Equal A Better Effect of Social Norm Information

In both studies, we found that group identity moderated the effect of social norm information on walking behavior. According to predictions of the TNSB, social norm information shared between members with a high group identity should make its effect more powerful [42]. However, we found that this type of effect of group identity was not reflected among males, while, among females, a higher group identity appeared to be a double-edged sword in terms of its effect on PA.

In particular, in study 1, we found that providing social norms with lower group identity (PA-inconsistent social norms) led to a reduced effect on walking in females, a finding which is consistent with the TNSB. This finding among females who were part of the PA-inconsistent group engaging in a low level of PA may be driven by 2 possible factors. First, we have to note the large disparity in performance between high-PA participants and low-PA participants in our study. For high-PA participants, the mean count of steps walked during the intervention stage was 11,997 (SD 3165), but for low-PA participants, the mean count of steps walked was 7868 (SD 2341). The later participants may have been likely to perceive themselves as outgroup members, thus weakening the effect of any social norm intervention. Second, these members may also have perceived a greater sense of upward social comparison, that is, comparison with peers who are relatively better off [71]. Thereafter, there may have been a subsequent loss of motivation to achieve a better ranking, leading to a reduction in walking. This effect is consistent with previous studies in which more upward comparison on social media platforms was associated with a decrease in self-esteem or well-being [72], as well as more depression or shame [73]. It has been suggested that interventions that lead to inappropriate types of social comparison, either upward or downward, may indeed be counter effective for one’s PA. Further studies should take the type of social comparison into account when considering the application of other potential factors (eg, age consistency [46]) to increase (or decrease) group identity theoretically.

In study 2, however, social norm information from a gender-consistent source, which should theoretically lead to a higher group identity, surprisingly led to a decrease in PA among females compared with PA in the self-monitoring control group. Although there are few interventions using gender-consistent social norms, our results are consistent with the finding in another health intervention study (providing gender-consistent social norm information is ineffective in reducing alcohol intake) [47]. One possible explanation might be that for females, sharing gender-consistent social norm information, that is, sharing gender-specific information, may provoke increased feelings associated with femininity and thus bring about concerns regarding their own social gender role. An alternative explanation could be that the separation of genders in ranking may unintentionally sharpen some undesirable social norms for females (eg, few females walk a lot), thus negatively impacting the intervention. For future interventions, we would argue that, at least in the context of promoting PA among females, tailoring social norm interventions to be gender consistent may not be advisable, since an unexpected side effect may conceal or even reverse the desired effect of the interventions.

### Implications

Based on WeChat groups and step ranking information as an ecological intervention, our study can provide new empirical evidence with high ecological validity regarding the effect of social norm information in promoting PA within the Chinese context. Our study contributes to the field of mHealth and PA interventions in 3 ways. First, by using a self-monitoring control group, our study avoided the high statistical error rate typically brought about by a mixed-strategy intervention. Second, our findings highlighted salient gender differences associated with the effects of social norm information and self-monitoring on PA. These findings suggest the necessity to take gender into account when designing mHealth interventions for Chinese users. Third, we advise caution in using group identity to promote PA. This is especially true for gender-specific manipulations, since the increase in identification with females may, in the context of promoting walking or general PA, conflict with the target behavior. 

Given the critical role of personalization in mHealth interventions, our results provide some useful insights into PA-targeted mHealth intervention projects in China. First, a gender-specialized intervention is essential to the goal of nudging walking behavior in China. For males, compared with a more traditional passive presentation of walking data, proactively pushing PA-related social norm information to users may be a more effective method for pedometer apps and other self-monitoring interventions. For example, WeRun or other mHealth apps could push a daily reminder containing the walking data of the friends of male users in order to maintain the initial beneficial effect of self-monitoring. However, among females, since social norm information did not have a beneficial effect on walking behavior, rather than adding extra social norm manipulations, maximizing the effect of self-monitoring may be a better approach. Female-targeted interventions should make full use of self-monitoring strategies. For example, they could push a daily personal step count to female users and track the overall walking step count record for each week. Second, nonessential gender-specific social norm information should be used with caution in PA interventions, since heightened gender identity may conflict with the target behavior, thus leading to an undesired outcome. Therefore, further mHealth interventions or smart devices should tailor their plans according to the gender of users; the provision of social norm information may be “the icing on the cake” for males but would be superfluous for females.

### Limitations

Several limitations of this research should be noted. All of our participants were Chinese college students, and this may limit the external validity and generalizability of our results. Although the main results were replicated across 2 studies to ensure their reliability, more empirical evidence is needed before generalizing our findings to other samples (eg, older adults) or other cultural backgrounds (eg, those with relatively weak gender stereotypes or gender gaps). Given that college students have a relatively high PA level (the average step count recorded by WeRun was 6932 steps/day in 2019 [74], while participants reached 7900 step/day in both our studies), further studies may investigate lower PA groups, such as office staff and retirees, to replicate our results.

In addition, previous studies have suggested that the effect of self-monitoring on PA can be attributed to the Hawthorne effect [26]. Although we aimed to compare the pure effect of self-monitoring and a social norm intervention, the nature of our study design means that we cannot rule out this possibility, that is, any effects observed may have been related to participants’ awareness that their walking activity was being monitored.

Finally, we did not provide sufficient empirical evidence to attribute our finding that females reduced their PA in the gender-consistent group to the presence of a backfire effect of high group identity on social norms. Other potential variables, such as trait competitiveness, may also impact the effect of social norm information. Further studies may benefit from controlling these variables to directly address this unexplained result.

### Conclusion

Gender and group identity are 2 moderators of the effect of social norm interventions on walking. In female Chinese college students, a higher sense of group identity does not guarantee a better effect of social norm information on PA, that is, PA-inconsistent or gender-consistent social norm information reduces walking in females. However, in male Chinese college students, social norm information and group identity do not show such an effect. Specifically, while walking may decrease with self-monitoring only, the inclusion of social norm information may help to keep the level of walking steady in males.

## Appendix

Multimedia Appendix 1 Additional analysis of the effect of self-monitoring and social norm information on walking behavior (study 1).

Multimedia Appendix 2 Additional analysis of the effect of self-monitoring and social norm information on walking behavior (study 2).

Multimedia Appendix 3 Descriptive information of social norms (study 1).

Multimedia Appendix 4 Descriptive information of social norms (study 2).

## Abbreviations

ANOVA: analysis of variance
LM model: linear mixed-effects model
mHealth: mobile health
PA: physical activity
PLM model: piece-wise linear mixed-effects model
TNSB: theory of normative social behavior
