# Correction: Gender-Specific Impact of Self-Monitoring and Social Norm Information on Walking Behavior Among Chinese College Students Assessed Using WeChat: Longitudinal Tracking Study

**DOI:** 10.2196/38221

**Published:** 2022-03-31

**Authors:** Yuepei Xu, Ling-Zi Yue, Wei Wang, Xiao-Ju Wu, Zhu-Yuan Liang

**Affiliations:** 1 CAS Key Laboratory of Behavioral Science Institute of Psychology Beijing China; 2 Department of Psychology, University of Chinese Academy of Sciences Beijing China; 3 Brigham and Women’s Hospital, Harvard Medical School Boston, MA United States

In “Gender-Specific Impact of Self-Monitoring and Social Norm Information on Walking Behavior Among Chinese College Students Assessed Using WeChat: Longitudinal Tracking Study” (J Med Internet Res 2021;23(12):e29167), one error was noted.

The foundation number of the National Natural Science Foundation of China was mistaken. In the originally published paper, under “Acknowledgments”, the foundation information was listed as follows:

This research was supported by Beijing Natural Science Foundation (BNSF, 9172019), the National Natural Science Foundation of China (NSFC, 7170111) …

This has been corrected to:

This research was supported by Beijing Natural Science Foundation (BNSF, 9172019), the National Natural Science Foundation of China (NSFC, 71471171) …

The correction will appear in the online version of the paper on the JMIR Publications website on March 31, 2022, together with the publication of this correction notice. Because this was made after submission to PubMed, PubMed Central, and other full-text repositories, the corrected article has also been resubmitted to those repositories.

