# Supplementary material for: Gender-Specific Impact of Self-Monitoring and Social Norm Information on Walking Behavior Among Chinese College Students Assessed Using WeChat: Longitudinal Tracking Study
Source: J Med Internet Res. 2021 Dec 7;23(12):e29167. doi: 10.2196/29167 (PMC8693203; doi:10.2196/29167)
Supplement: Multimedia Appendix 1 [file jmir_v23i12e29167_app1.docx]

### Multimedia Appendix 1. Additional analysis of the effect of self-monitoring and social norm information on walking behavior (study 1).

Considering that the ranking position may impact the effect of social norms, we added ranking position as a new covariate. For the coding of ranking position, in order to control for the potential impact of the number of people participating in the ranking, we rescaled the raw ranking positions (i.e., 1^st^, 2^nd^, 3^rd^ …) into a percentile in each group respectively. For the control group, since there were no rankings assigned to them, all ranking positions were encoded as -1. As such, the mixed linear model for the intervention period was set up with group, time (range from 1 to 14) and their interactions as predictors, with BMI and ranking position as covariates. See Table S1 for detailed information.

For males, we found that the interaction between time and group was marginally significant, *F*(2, 679.22) = 2.82, *P* = .06. In the self-monitoring group, we observed a significantly negative slope (*p* = .04), suggesting that the step count of males in this group decreased during the intervention period, while both the consistent and inconsistent interventions were able to arrest this decreasing trend. Moreover, the ranking position was a marginally significant covariate of walking behavior, *F* (1, 726.92) = 2.77, *P* = .10, suggesting that a higher ranking motivated males to walk more (slope = – 11.31, *SE* = 6.79).

For females, we observed that the interaction between time and group was significant, *F*(2, 791.65) = 3.70, *P* = .03. In the inconsistent intervention group, we found a significant negative slope (*p* = .02) for those exposed to the inconsistent intervention such that there was a decrease in the step count during this period. This effect was not present in either the self-monitoring or PA-consistent intervention group. Moreover, for females, we found that BMI was a significant covariate of walking behavior, *F* (1, 57.70) = 4.28, *P* = .04, suggesting that a higher BMI motivated female to walk more (slope = 326.25, *SE* = 157.69).

Table S1. Intercept and slopes of the ranking position added mixed linear model for the control and intervention groups in study 1.

| Gender | Group (*n*) | Intercept | Slope | *SE* | *P* | Conditional *R*^2^ |
| --- | --- | --- | --- | --- | --- | --- |
| Male  (*n*=54) | Self-monitoring (15) | 8030.14 | -144.84 | 71.52 | .04 | 0.29 |
|  | PA-consistent Intervention (21) | 8181.69 | 49.19 | 61.45 | .42 |  |
|  | PA-inconsistent Intervention (18) | 9747.62 | 63.98 | 66.48 | .34 |  |
| Female (*n*=63) | Self-monitoring (22) | 8065.24 | 81.11 | 57.38 | .16 | 0.22 |
|  | PA-consistent Intervention (19) | 7844.15 | 17.46 | 62.64 | .78 |  |
|  | PA-inconsistent Intervention (22) | 7988.55 | -135.20 | 58.15 | .02 |  |
