# Supplementary material for: Gender-Specific Impact of Self-Monitoring and Social Norm Information on Walking Behavior Among Chinese College Students Assessed Using WeChat: Longitudinal Tracking Study
Source: J Med Internet Res. 2021 Dec 7;23(12):e29167. doi: 10.2196/29167 (PMC8693203; doi:10.2196/29167)
Supplement: Multimedia Appendix 2 [file jmir_v23i12e29167_app2.docx]

### Multimedia Appendix 2. Additional analysis of the effect of self-monitoring and social norm information on walking behavior (study 2).

To compare the slopes produced during the intervention period, we performed an intervention-focused mixed linear model for each gender, with group, time (range from 1 to 15) and their interactions as predictors. Detailed results from the mixed linear model during the intervention period are shown in Table S1.

Table S1. Intercept and slopes of the intervention-focused mixed linear model for the control and intervention groups (study 2).

| Gender | Group | Intercept | Slope | *SE* | *P* | Conditional *R*^2^ |
| --- | --- | --- | --- | --- | --- | --- |
| Male  (*N*=88) | Self-monitoring(*N*=23) | 7904.37 | -30.81 | 44.19 | .49 | 0.28 |
|  | Gender-consistent Intervention(*N*=33) | 8570.34 | -7.46 | 36.79 | .84 |  |
|  | Gender-inconsistent Intervention(*N*=32) | 7829.62 | 51.08 | 37.46 | .17 |  |
| Female  (*N*=92) | Self-monitoring (*N*=26) | 7357.30 | 52.58 | 41.44 | .20 | 0.20 |
|  | Gender-consistent Intervention(*N*=34) | 8079.34 | -41.25 | 35.81 | .25 |  |
|  | Gender-inconsistent Intervention(*N*=32) | 7957.84 | 23.58 | 37.13 | .53 |  |

Considering that the ranking position may impact the effect of social norms, we additionally added ranking position as a new covariate for Study 2. The coding of ranking position was the same as the additional analysis for Study 1. Briefly, with ranking position as a covariate, the significance of results remained the same and ranking position was not a significant covariate. See detailed information in Table S2.

Table S2. Intercept and slopes of the ranking position added mixed linear model for the control and intervention groups in study 2.

| Gender | Group | Intercept | Slope | *SE* | *P* | Conditional *R*^2^ |
| --- | --- | --- | --- | --- | --- | --- |
| Male  (*N*=88) | Self-monitoring(*N*=23) | 7946.47 | -30.81 | 44.19 | .49 | 0.28 |
|  | Gender-consistent Intervention(*N*=33) | 8555.52 | -9.00 | 37.22 | .81 |  |
|  | Gender-inconsistent Intervention(*N*=32) | 7814.55 | 49.68 | 37.82 | .19 |  |
| Female  (*N*=92) | Self-monitoring (*N*=26) | 7193.06 | 52.64 | 41.51 | .21 | 0.19 |
|  | Gender-consistent Intervention(*N*=34) | 8146.24 | -35.07 | 36.27 | .33 |  |
|  | Gender-inconsistent Intervention(*N*=32) | 8018.64 | 29.74 | 37.57 | .43 |  |
