# Supplementary material for: Gender-Specific Impact of Self-Monitoring and Social Norm Information on Walking Behavior Among Chinese College Students Assessed Using WeChat: Longitudinal Tracking Study
Source: J Med Internet Res. 2021 Dec 7;23(12):e29167. doi: 10.2196/29167 (PMC8693203; doi:10.2196/29167)
Supplement: Multimedia Appendix 3 [file jmir_v23i12e29167_app3.docx]

### Multimedia Appendix 3. Descriptive information of social norms (study 1).

We conducted a mixed-effects linear model with the stage as a within-group factor, group and gender as two between-group factors, BMI as a covariate, and participants’ ID as a random intercept. Since the linear model did not find any significant effects of group, we split the descriptive results of social norms by gender and stage (see Table S1).

For injunctive social norm, the only significant difference we found was in the female group between the baseline (5.46±1.12) and follow-up (5.83±0.77) stage, *P* = .004. For descriptive gender norms, all of the pairwise comparisons of different experimental stages were significant (see Table S1), thus there was a significant increasing trend as stages progressed for both genders. In other words, participants perceived and estimated more and more students walked over 6000 steps/day progressively.

Table S1. Results for both injunctive and descriptive social norm for each gender during the baseline, intervention and follow-up stages in study 1 (*M* ± *SD*) ^a-c^

| Gender | Social Norm | Experiment Stage | | | *P*^1^ | *P*^2^ | *P*^3^ |
| --- | --- | --- | --- | --- | --- | --- | --- |
|  |  | Baseline | Intervention | Follow-up |  |  |  |
| Male  (*n* = 54) | Injunctive norms | 5.76±0.75 | 5.64±0.80 | 5.62±0.94 | .83 | .77 | .99 |
|  | Descriptive male norms | 39.68±19.85 | 48.49±18.87 | 54.29±17.37 | <.001 | <.001 | .004 |
|  | Descriptive female norms | 34.03±19.14 | 42.31±19.27 | 48.08±17.23 | <.001 | <.001 | .002 |
| Female  (*n* = 63) | Injunctive norms | 5.46±1.12 | 5.65±0.93 | 5.83±0.77 | .35 | .004 | .16 |
|  | Descriptive male norms | 44.12±19.45 | 53.56±16.32 | 58.64±16.29 | <.001 | <.001 | .01 |
|  | Descriptive female norms | 33.19±18.60 | 42.64±16.36 | 47.69±18.01 | <.001 | <.001 | .03 |

^a^*P* refers to the results of pairwise comparisons between each two experiment stages.

^b^ *P*^1^: baseline and intervention; *P*^2^: baseline and follow-up; *P*^3^: intervention and follow-up.

^C^All *P*s were adjusted by Tukey method.
