# Supplementary material for: Gender-Specific Impact of Self-Monitoring and Social Norm Information on Walking Behavior Among Chinese College Students Assessed Using WeChat: Longitudinal Tracking Study
Source: J Med Internet Res. 2021 Dec 7;23(12):e29167. doi: 10.2196/29167 (PMC8693203; doi:10.2196/29167)
Supplement: Multimedia Appendix 4 [file jmir_v23i12e29167_app4.docx]

### Multimedia Appendix 4. Descriptive information of social norms (study 2).

Table S1 lists the descriptive results for the injunctive norms and descriptive gender norms for each gender and experimental stage.

Table S1. Injunctive and descriptive social norms of different gender during baseline, intervention and follow-up stage in study 2(*M* ± *SD*) ^a-c^

| Gender | Social Norm | Experiment Stage | | | *P*^1^ | *P*^2^ | *P*^3^ |
| --- | --- | --- | --- | --- | --- | --- | --- |
|  |  | Baseline | Intervention | Follow-up |  |  |  |
| Male  (*n* = 88) | Injunctive norms | 5.33±1.21 | 5.53±0.86 | 5.48±0.79 | .21 | .43 | .89 |
|  | Descriptive male norms | 35.78±20.49 | 41.23±17.88 | 48.99±19.45 | .05 | <.001 | <.001 |
|  | Descriptive female norms | 26.78±17.80 | 33.67 ±17.95 | 41.52±18.69 | .01 | <.001 | <.001 |
| Female  (*n* = 92) | Injunctive norms | 5.37±1.02 | 5.53±1.05 | 5.56±0.94 | .42 | .18 | .87 |
|  | Descriptive male norms | 47.25±20.45 | 51.78±20.73 | 58.54±19.47 | .11 | <.001 | .004 |
|  | Descriptive female norms | 33.49±18.43 | 40.38±19.56 | 46.52±19.28 | .002 | <.001 | .004 |

^a^*P* refers to the results of pairwise comparisons between each two experiment stages.

^b^ *P*^1^: baseline and intervention; *P*^2^: baseline and follow-up; *P*^3^: intervention and follow-up.

^C^All *P*s were adjusted by Tukey method.
